# The Interplay between Endogenous and Foodborne Pro-Oxidants and Antioxidants in Shaping Redox Homeostasis

**DOI:** 10.3390/ijms25147827

**Published:** 2024-07-17

**Authors:** Patrycja Jakubek, Karol Parchem, Mariusz R. Wieckowski, Agnieszka Bartoszek

**Affiliations:** 1 Department of Food Chemistry, Technology and Biotechnology, Faculty of Chemistry, Gdańsk University of Technology, 80-233 Gdańsk, Poland; karol.parchem@pg.edu.pl; 2 Laboratory of Mitochondrial Biology and Metabolism, Nencki Institute of Experimental Biology, Polish Academy of Sciences, 02-093 Warsaw, Poland; m.wieckowski@nencki.edu.pl

**Keywords:** redox-active substances, redox homeostasis, reactive oxygen species, redox chemistry

## Abstract

Oxidative stress has been known about in biological sciences for several decades; however, the understanding of this concept has evolved greatly since its foundation. Over the past years, reactive oxygen species, once viewed as solely deleterious, have become recognized as intrinsic components of life. In contrast, antioxidants, initially believed to be cure-all remedies, have failed to prove their efficacy in clinical trials. Fortunately, research on the health-promoting properties of antioxidants has been ongoing. Subsequent years showed that the former assumption that all antioxidants acted similarly was greatly oversimplified. Redox-active compounds differ in their chemical structures, electrochemical properties, mechanisms of action, and bioavailability; therefore, their efficacy in protecting against oxidative stress also varies. In this review, we discuss the changing perception of oxidative stress and its sources, emphasizing everyday-life exposures, particularly those of dietary origin. Finally, we posit that a better understanding of the physicochemical properties and biological outcomes of antioxidants is crucial to fully utilize their beneficial impact on health.

## 1. Oxidative Stress—Definition and Evolution of the Concept

**Figure 1:**
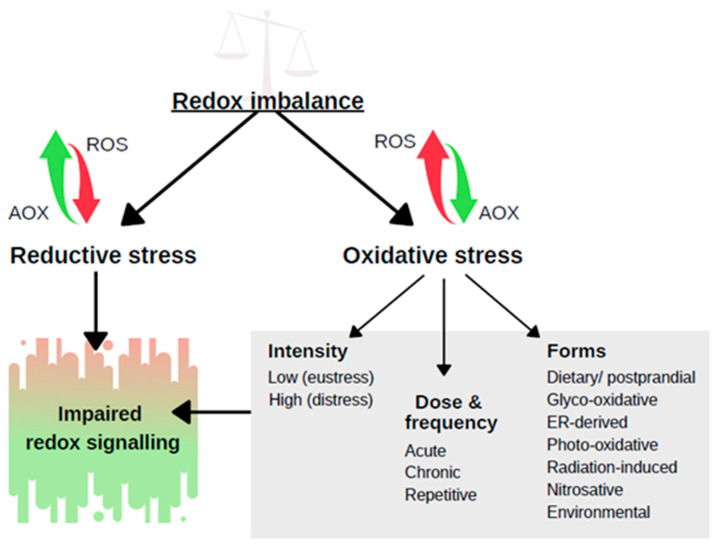
The concept of oxidative stress and related terms. Abbreviations: AOX, antioxidants; ROS, reactive oxygen species; ER, endoplasmic reticulum.

In a biological sense, stress was first recognized by Hans Selye, an endocrinologist working on hormonal changes in rats, who described it in a letter published in *Nature* in 1936 as a “*syndrome that appears in response to damage*” [1]. In a biological or medical sense, the term oxidative stress began to appear in scientific literature no sooner than in the 1970s; however, it was not defined until 1985 when Helmut Sies published his book entitled simply *Oxidative Stress* [2,3]. The term oxidative stress was initially understood as “a disturbance in the pro-oxidant–antioxidant balance in favour of the former” [3], and at that time researchers focused mainly on oxidative damage and its role in disease development. As new discoveries were demonstrating the inefficacy of antioxidant-based therapies on one hand [4,5,6,7] and the previously overlooked physiological roles of reactive oxygen species (ROS) on the other [8], the limitations and inadequacy of the above definition of oxidative stress became evident [9]. With time, antioxidants and ROS were no longer considered solely “the good guys” and “the bad guys”, respectively. Research interests have shifted from ROS-induced damage to biomolecules to redox signalling and regulation. The understanding of oxidative stress was evolving concurrently and now it is defined as “*an imbalance between oxidants and antioxidants in favour of the oxidants, leading to the disruption of redox signalling and control and/or molecular damage*” (Figure 1) [10].

In general, ROS are generated as by-products of aerobic metabolism, which at appropriate physiological levels act as signalling messengers and regulators of numerous cellular processes, especially those requiring energy, such as cell proliferation, adhesion, differentiation, survival, or death [11]. Therefore, oxidative stress is further classified based on its intensity as eustress, corresponding to the level of ROS necessary to maintain physiological cellular functions, and distress, referring to an excessive oxidative burden that results in cellular damage and dysfunction [12]. Concerning the dose and frequency of oxidative challenge, stress can be divided into three categories: acute, chronic, and repetitive, all of which can play a role in oxidative stress adaptation [13]. Furthermore, depending on the trigger, oxidative stress can take the form of dietary postprandial, photo-oxidative, radiation-induced, nitrosative, glyco-oxidative, endoplasmic reticulum (ER)-derived, or environmental stress [10]. The opposite processes, that is, depletion of ROS and surplus of reducing agents, push the redox equilibrium towards reductive stress [14]. Reductive stress can be characterized as a situation in which the accumulation of endogenous antioxidants (e.g., glutathione, abbreviated as GSH), reducing equivalents (e.g., NAD(P)H/NAD(P)+), or exogenous antioxidants affects cellular control or regulation of redox-related processes through the Nrf2–Keap1 system and other redox-related factors [15]. Nevertheless, in contrast to oxidative stress, the concept of reductive stress has not been well-established [16]. The reader can refer to the review by Kuczyńska et al. [15] for further information on the role of reductive stress in disease development.

## 2. ROS and Their Endogenous Production

Although the presence of free radicals in biological systems was first observed in the 1950s, it was not until the mid-1970s when it became evident that they were not the only molecules participating in free radical chain reactions [17,18]. Therefore, the expression “reactive oxygen species” was introduced to collectively name all oxygen-derived molecules of free-radical or non-radical nature formed upon the reduction of molecular oxygen. The most abundant ROS include superoxide radical ($O_{2}^{•-}$), hydroxyl radical (${\mathrm{HO}}^{•}$), and hydrogen peroxide ($H_{2}O_{2}$). There are also many other reactive species present in the cellular redox network, which include, among others, nitrogen- and sulphur-centred entities that, by analogy, are called RNS and RSS, respectively. Among RNS, which also contain oxygen atoms, and thus can be treated as ROS, nitric oxide (NO) and peroxynitrate (${\mathrm{ONOO}}^{-}$) are of utmost importance. The subject of RNS and RSS is beyond the scope of this review; however, the reader can refer to the works authored by Carmen Martínez et al. [19], Gruhlke and Slusarenko [20], and Olson [21] for comprehensive information.

**Figure 2:**
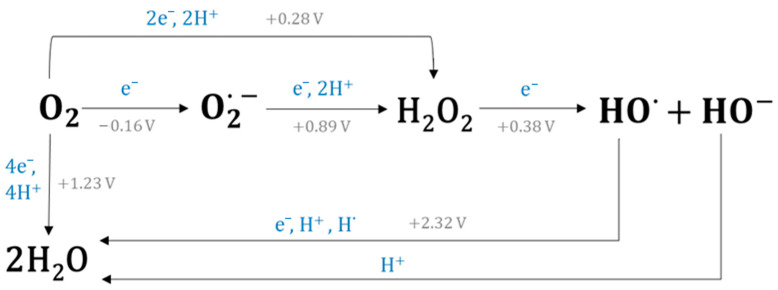
Electrochemical parameters and electron transfer during the formation of reactive oxygen species. The formal reduction potential values (at pH 7) were adapted from Li et al. (2019) [29].

Despite the general belief that mitochondria are the main cellular source of ROS, definite experimental evidence to support this claim is missing, as discussed by Brown and Borutaite [22]. Nevertheless, there is evidence that mitochondria may be a significant source of ROS under various pathological conditions. Most mitochondrial ROS are generated during oxidative phosphorylation due to electron leakage from the electron transport chain (ETC), particularly at the stage where molecular oxygen undergoes one-electron reduction to $O_{2}^{•-}$. As many as seven sites of $O_{2}^{•-}$ production have been identified in mammalian mitochondria: the ubiquinone-binding sites in complex I (site IQ) and complex III (site IIIQo) of the ETC, glycerol-3-phosphate dehydrogenase, the flavin in complex I (site IF), the electron transfer flavoprotein:Q oxidoreductase of fatty acid (FA) β-oxidation and pyruvate or 2-oxoglutarate dehydrogenases; however, only two of them (complexes I and III of ETC) are recognized to be the major ones [23]. It has been estimated that 1–4% of the molecular oxygen consumed by mitochondrial respiration is converted to $O_{2}^{•-}$, although lower values of approximately 0.15% have also been reported [24,25]. These values were assessed under in vitro conditions in isolated mitochondria; therefore, extrapolation to in vivo situations must be carried out with caution [26]. Still, $O_{2}^{•-}$ is considered to be the most abundant by-product of cellular metabolism, from which other ROS may originate. Dismutation of $O_{2}^{•-}$ (spontaneous or enzymatic) gives rise to $H_{2}O_{2}\;$which can form ${\mathrm{HO}}^{•}$ by the acquisition of one more electron (e.g., in the course of metal-catalysed Fenton reaction). The latter is the most reactive radical and least-specific ROS. Owing to its high reduction potential, it reacts rapidly with any molecule present in its closest neighbourhood [27]. Finally, upon the addition of one proton and one electron, ${\mathrm{HO}}^{•}$ is reduced to a water molecule (Figure 2) [28]. In vivo, this reaction occurs through the abstraction of the hydrogen atom from the lipid or protein structures, thereby initiating chain radical reactions involving these biomolecules.

Peroxisomes constitute another site of endogenous ROS generation resulting from oxidative metabolism of, e.g., FAs, glyoxylate, amino acids as well as phospholipid biosynthesis, which takes place there. These organelles contain multiple enzymes that produce reactive forms of oxygen and nitrogen; xanthine oxidoreductase (XOR) is considered the major one. XOR is a rate-limiting enzyme in purine catabolism as it catalyses the oxidation of hypoxanthine to xanthine, followed by further oxidation to uric acid. In the course of these reactions, XOR generates $H_{2}O_{2}$ and $O_{2}^{•-}$ as by-products [30].

In the ER, a relatively oxidizing environment is maintained in the lumen, as here $H_{2}O_{2}$ is produced and utilized in the processes of oxidative protein folding [31]. Disturbances in ER redox homeostasis may lead to the accumulation of unfolded proteins. This results in ER stress, in which excessive ROS generation causes oxidative stress [32]. Besides this, $H_{2}O_{2}$ and $O_{2}^{•-}$ can also be generated during the oxidative metabolism of xenobiotics, haem, and FAs involving microsomal cytochrome P450-dependent monooxygenases and cytochrome *b*5 [33]. Indeed, in the rat liver, the microsomal cytochrome P450-dependent monooxygenase system has been suggested to be the greatest and most active source of ROS, as up to 45% of $H_{2}O_{2}$ was reported to be produced by this enzymatic system [34].

Plasma membrane-bound nicotinamide adenine dinucleotide phosphate (NADPH) oxidases (NOXs) constitute another major producer of ROS, whose generation is established as the primary and probably the sole function of these enzymes [35]. Both phagocytic and nonphagocytic isoforms of NADPH oxidases (NOX1-3) mediate the release of $O_{2}^{•-}$ as a side product of electron transfer from NADPH to molecular oxygen. NOXs present in phagocytic cells release ROS in response to fungal, bacterial, and viral infections to eradicate pathogenic microorganisms [36,37]. Nonphagocytic NOXs generate lower levels of $O_{2}^{•-}$ than those present in phagocytic cells, even under pathological conditions [38]. The latter NOXs are expressed in various cell types, including fibroblasts, vascular endothelial cells, neurons and skeletal muscle myocytes, where ROS are involved in intra- and intercellular communication, redox signalling and proper cell function [39,40,41,42]. ROS production in response to inflammatory processes also occurs via alternative pathways involving lipoxygenase (LOX)- and cyclooxygenase (COX)-mediated oxidation of arachidonic acid released from membrane phospholipids to produce prostaglandins, leukotrienes, and thromboxanes [43]. Arachidonic acid and its metabolites have also been reported to induce ROS generation by NOXs [44,45,46].

Nitric oxide synthase (NOS) constitutes an important source of NO formed upon the conversion of L-arginine to L-citrulline. Under certain conditions, NOS can also generate $O_{2}^{•-}$, $H_{2}O_{2}$, and ${\mathrm{ONOO}}^{-}$. In mammals, there are four distinct isoforms of NOS: inducible (iNOS), endothelial (eNOS), neuronal (nNOS), and mitochondrial (mtNOS) [47,48]. iNOS is stimulated in response to inflammation and infection [49]. eNOS generates NO, which is crucial for cardiovascular health because it regulates blood pressure, vascular tone, and platelet function [47,50]. nNOS, which is constitutively expressed in the central nervous system, has been proposed to modulate synaptic plasticity and neuronal signalling [51]. It has been suggested that mtNOS controls oxygen consumption by competitively inhibiting mitochondrial cytochrome oxidase. Such modulation is regarded as transient, as long as NO is produced at low concentrations. However, higher concentrations of this compound can lead to enhanced ROS generation through its inhibitory impact on the ETC [48].

## 3. Exposome-Derived (Exogenous) ROS

The term exposome, as proposed in 2005 by Christopher P. Wild, includes all environmental exposures experienced during a person’s life (including lifestyle factors) from the prenatal period onwards [52,53]. This definition encompasses all external and internal (but non-genetically determined) exposures that can be divided into three groups: (1) general external exposome, which is an inseparable part of human life, such as the urban–rural environment, and climate factors; (2) specific external exposome, which refers to lifestyle-based choices such as diet, physical activity, and cigarette smoking as well as professional occupation, individual health-related aspects (infections and medical drugs) and radiation exposure; and (3) internal exposome, which involves internal biological factors, such as metabolism, gut microbiota, inflammatory processes, circulating hormones and redox status (Table 1) [54,55]. In this subsection, the impact of selected exogenous factors on ROS formation is discussed (Table 1) with special emphasis on diet-related inducers of oxidative stress.

### 3.1. Diet-Induced Oxidative Stress

With an average surface of 32 m^2^, the gastrointestinal (GI) tract is the largest area in a human organism that is constantly exposed to the external environment. This makes the human gut notoriously challenged by numerous pro-oxidants, both the exogenous ones swallowed with food and those arising during food intestinal passage [61,62]. Most pro-oxidants in the GI tract are of dietary origin; hence, the term nutritional oxidative stress was defined by Helmut Sies as “*an imbalance between the pro-oxidant load and the antioxidant defence as a consequence of excess oxidative load or of inadequate supply of the organisms with nutrients*” [63]. A subform of nutritional oxidative stress is postprandial oxidative stress, which “*is characterised by an increased susceptibility of the organism toward oxidative damage after consumption of a meal rich in lipids and/or carbohydrates*” [63]. Thus, on the one hand, modified macronutrients, such as oxidized lipids, may be formed during food production, processing, storage or final preparation by the consumers, and be ingested with a meal. On the other hand, dietary macronutrients can be targets for oxidative modifications during gastrointestinal digestion [64] and after their intestinal absorption, for example, in the circulatory system. Importantly, all of these forms could affect the oxidative damage of circulating lipoproteins, contributing to atherosclerosis development [65].

A perfect example that fulfils both the above-mentioned scenarios is the Maillard reaction (MR). MR refers to non-enzymatic browning reactions between reducing sugars and compounds with free amino groups that occur upon thermal processing and storage of food. MR consists of three stages: early (initiation), intermediate (propagation) and late (advanced) [66]. In brief, in the early stage, electrophilic carbonyl groups of reducing sugars react with nucleophilic amino groups of amino acids, peptides and proteins to form stable intermediate compounds named Amadori rearrangement products (ARPs) in the case of reducing aldoses or Heyns rearrangement products in the case of reducing ketoses [67]. These intermediate compounds are transformed into more reactive carbonyls (mostly glyoxal, methylglyoxal and diacetyl) during the intermediate stage [68]. In the late stages, carbonyl compounds react with each other and with amino groups in a series of chemical reactions, including condensation, polymerization, degradation and cyclization, forming favourable sensory attributes such as a brown colour as well as appetizing aromas and flavours (e.g., bakery products, roasted coffee) [66]. Brown pigmentation can be attributed mainly to melanoidins, which are non-volatile, high-molecular-weight heterogeneous polymers formed in the late stages of MR at high temperatures. Among MR products, aminoreductones, melanoidins, and ARPs possess antioxidant properties that delay or inhibit lipid oxidation in food by their ability to chelate transition metals (Cu^2+^, Fe^2+^), scavenge oxygen radicals, and inhibit polyphenol oxidase, thus preventing enzymatic browning of foods of plant origin [69,70]. MR products have also been shown to exhibit antioxidant effects in vitro. Specifically, melanoidins extracted from biscuits and bread crust protected human hepatocarcinoma HepG2 and human intestinal Caco-2 cell lines, respectively, from oxidative challenge [71,72]. Wistar rats fed with biscuits, a rich source of MR products, showed increased antioxidant activity and reduced levels of lipid oxidation in the serum [73]. Food extracts rich in MR products have been shown to inhibit copper-induced oxidation of LDL in human serum tested under in vitro conditions. This in vitro assessment was followed by a pilot intervention study involving eight healthy volunteers whose diet was supplemented with products rich in MR products for one week. Again, the obtained results implied that thermally processed foods rich in MR products (sterilized milk, pretzel sticks, dark chocolate, farmhouse bread, roasted or fired meat, dark beer and coffee) increase antioxidant activity in human serum tested ex vivo [74]. However, another study reported that a diet rich in MR products did not affect markers of oxidative damage or antioxidant defence status in 18 healthy male adolescents [75]. In contrast to these findings, other reports have shown that MR products have a detrimental impact on redox homeostasis. MR products (prepared under laboratory conditions by heating a mixture of amino acids and reducing sugars) were shown to cause a significant increase in the level of DNA damage and decrease in the level of GSH and activity of GR and CAT in human lymphocytes [76]. These results are in line with data showing a positive association between a higher intake of ultra-processed foods, which are rich in MR products and higher DNA damage levels in healthy adolescents from Iran [77]. High intake of ultra-processed foods has also been linked to enhanced systemic oxidative stress [78]. On the other hand, coffee is an example of a product, which despite being highly processed, contains numerous antioxidants that are known to contribute to health-promoting properties of this beverage [79,80]. Hence, the impact of MR products on redox status should not be judged without the context of the food products containing them. To further complicate this subject, MRs increase the amount of reactive carbonyl compounds, which can lead to carbonyl stress and ultimately aid in the generation of advanced glycation end products (AGEs) via glycoxidation of free amino groups in proteins [81]. AGEs have also been shown to enhance the generation of reactive oxygen and nitrogen species in human keratinocytes [82]. More information on the relationship between AGEs and oxidative stress in disease development can be found in the recent review by Peng et al. [68].

One vivid example of pro-oxidative nutrition is the so-called Western diet (WD), which is currently widespread in the majority of industrialized countries [83]. WD is characterized by high-calorie meals rich in refined carbohydrates and lipids, but poor in fibre, vitamins, minerals, and other bioactive compounds. It has been generally accepted that over recent decades a Westernized lifestyle has led to a dramatic increase in the occurrence of chronic non-communicable diseases such as obesity, metabolic syndrome, type 2 diabetes (T2D), various types of cancer, neurodegenerative diseases, and depression [84,85]. These diseases are also associated with impaired redox homeostasis [86,87]. WD may contribute to oxidative stress in several ways. The first mechanism is related to the dysregulation of FA homeostasis resulting from prolonged nutrient overconsumption and exceeding the capacity of adipose tissue to store fat (either due to an inability to generate sufficient new adipocytes or an inability to further expand the existing ones) [88]. This phenomenon, combined with a high-fat diet, results in the increased plasma levels of non-esterified FAs (NEFA) and their accumulation in non-adipose tissues, such as liver, pancreas, skeletal and heart muscles, or kidneys [89]. To handle excess NEFAs, cellular metabolism requires efficient disposal strategies, including the esterification of NEFAs into triacylglycerols (TGs) and enhanced β-oxidation. However, in the case of non-adipose tissues, the cellular capacity for NEFAs storage is limited, while enzymes required for β-oxidation may become depleted. Consequently, it may lead to the accumulation of intermediates of lipid metabolism, such as diacylglycerols (DGs) or ceramides, which can disturb other metabolic pathways, contributing to the development of lipotoxic effects [89]. Lipotoxicity is associated with the induction of ER stress, an increase in cell-autonomous inflammatory responses [89], as well as insulin resistance, which can lead to the development of metabolic syndrome, T2D, or metabolic dysfunction-associated steatotic liver disease (MASLD). One of the mechanisms involved in insulin resistance is inhibition of glucose transport and phosphorylation, leading to reduced glucose oxidation rates and glycogen synthesis. Furthermore, NEFA and FA intermediates, such as DGs, ceramides, and acylcarnitines, can alter insulin-signalling pathways, including protein kinase B or phosphatidylinositol 3-kinase kinase [89]. Regarding the mechanisms of insulin resistance in pancreatic β-cells, the concept of glucolipotoxicity has also been proposed, in which simultaneous cell exposure to high levels of palmitic acid and glucose leads to induction of oxidative stress, loss of insulin secretory function, apoptosis and β-cell death [90]. Furthermore, lipotoxicity can also lead to mitochondrial dysfunction, including impaired electron transport chain (ETC) activity, reduced ATP production, and increased electron leakage, resulting in elevated ROS formation as well as enhancement of mitochondrial oxidative stress, which may induce modifications of biomolecules such as lipids, proteins, and DNA [88]. However, recently, some contradictory findings have also been reported, that is, neither mitochondrial dysfunction nor noticeable mitochondrial oxidative stress was found in the livers of mice fed with WD and in liver biopsies of patients with MASLD [91,92,93]. These reports point to the fact that one must be cautious when extrapolating in vitro to in vivo situations [93].

Second, the consumption of calorically rich ultra-processed foods is postulated to promote oxidative stress and low-grade inflammation [94]. Ultra-processed foodstuffs are usually characterized by a high content of simple sugars, whose ingestion results in a rapid rise in blood glucose levels [95]. Importantly, in the oral glucose tolerance test, the plasma antioxidant status, measured as the total radical trapping capacity of the plasma, decreased significantly in healthy and diabetic subjects [96]. Another study indicated that glucose concentration was found to correlate with postprandial low-density lipoprotein (LDL) oxidation [97]. This indicates that hyperglycaemia induces free radical formation and contributes to NO oxidation, and a reduction in its concentration leads to endothelial dysfunction [98]. Ultimately, this results in imbalanced vascular homeostasis, leading to a prothrombotic, proinflammatory, and less-compliant blood vessel wall [99]. Epidemiological studies have also indicated a positive correlation between the dietary glycaemic index (GI) and the concentration of C-reactive protein, providing evidence for a positive association between GI and low-grade inflammation [98]. WD is also characterized by the overconsumption of vegetable oils rich in n−6 polyunsaturated FAs (PUFAs) and an insufficient intake of n−3 PUFAs [84,100,101]. To illustrate this aspect, it should be mentioned that the recommended ratio of n−6 to n−3 PUFAs in humans is less than 4, while for WD, this ratio can range from 10:1 to as much as 20:1 [102]. Because of the role of linoleic acid (LA, 18:2, n−6) as a precursor of arachidonic acid (AA, 20:4, n−6), and the fact that both can be substrates for the synthesis of pro-inflammatory oxylipins, it is widely considered that elevated consumption of n−6 PUFAs and low intake of n−3 PUFAs promote systematic, low-grade inflammation [103]. However, it should be mentioned that the available evidence does not support this hypothesis anymore [98]. For example, a 16-year follow-up study performed by Zhuang et al., in which 521,120 participants aged from 50 to 71 years were involved, concluded that total mortality and mortality related to cardiovascular diseases (CVDs) were significantly reduced in association with increasing intake of marine n−3 PUFAs such as eicosapentaenoic acid (EPA) and docosahexaenoic acid (DHA), total n−6 PUFA, and LA, while the n−3 to n−6 ratio was irrelevant to the same parameters [104]. On the other hand, PUFAs, owing to their susceptibility to oxidation, could be precursors of hazardous substances such as 4-hydroxy-hexenal (4-HHE). Therefore, questions may arise regarding the safety of PUFA ingestion and the potential paradox of their health benefits and potential harmful effects [105]. As already mentioned, lipid peroxidation products can be formed during food/supplement production and/or storage, as well as in vitro, through endogenous oxidative processes. Most studies have reported increased in vitro plasma-lipid oxidation following supplementation with fish oils [106]. For example, in a study performed by Calzada et al., in which subjects (*n* = 12) were supplemented for two weeks with DHA (0.8 or 1.6 g/day), a significant level of 4-HHE (a peroxidation product specifically derived from DHA) was observed; however, the efficiency of the conversion of DHA to 4-HHE was estimated to be 0.01%, whereas the concentration of 4-hydroxy-nonenal (4-HNE) (derived from the peroxidation of n−6 FAs that were not supplemented) did not change [106]. In another study, individuals who consumed a sunflower oil-based diet (rich in PUFAs) were reported to have increased total levels of DNA-malondialdehyde (MDA) adducts inwhite blood cells compared with those who consumed a rapeseed oil-based diet rich in MUFAs [107]. On the other hand, there are also studies showing that fish oil or n−3 PUFAs did not increase the susceptibility of plasma lipids to oxidation, and even decreased it [106]. Such discrepancies may be related to numerous factors, including differences in the populations studied, the composition and dosage of the n−3 PUFAs supplement, and its quality, including oxidative status, duration of studies, and methodologies used. Therefore, it is postulated that supplementations of n−3 PUFAs with the recommended doses of 0.5–1 g/day do not significantly affect the induction of oxidative stress and do not cause cytotoxic effects and carcinogenic potential [105], as long as the supplements meet quality requirements, including oxidation marker levels. Moreover, such a dose seems to be sufficient to achieve the health-promoting effects of ingestion n−3 PUFAs. For example, the European Food Safety Authority (EFSA) recommends a daily intake of 250 mg of EPA and DHA to reduce the risk of heart disease in healthy adults (primary prevention), while the American Heart Association (AHA) recommends a daily intake of 1 g of EPA and DHA for the secondary prevention of coronary heart disease (CHD) [108].

Third, ultra-processed foods, characteristic of the WD, could be a significant source of modified macronutrients, and their overconsumption may promote low-grade inflammation and increase oxidative stress. For example, it was estimated that the average daily consumption of lipid oxidation products by humans includes 420 mg of lipid hydroperoxides, 105 mg of lipid epoxides, 4.2 mg of acrolein, 233 μg of MDA, 38 μg of HNE, and 4.2 μg of HHE [109,110]. In general, lipid peroxidation products are recognized as harmful substances, and their chronic and/or excessive consumption may lead to the development of pathological conditions such as atherosclerosis, liver dysfunction, neurological diseases, T2D, or cancer [111]. However, it has been postulated that some studies suggesting an increased risk of pathological state development after consumption of thermally oxidized lipids are conducted using animal models fed with amounts of oxidized lipids, which are unattainable for humans, even in the case of consuming meals rich in fried food. Therefore, drawing conclusions regarding human diseases mediated by dietary oxidized lipids could be inconclusive and limited [112]. In particular, substances that exhibit positive effects may also appear in the dietary lipid peroxidation products. For example, azelaic acid (saturated dicarboxylic acid), which is formed upon decomposition of 13-hydroxyoctadecadienoic acid to 9-oxononanoic acid followed by its oxidation, was found to induce apolipoprotein A1 (ApoA1) secretion and paraoxonase 1 (PON1) activity and suppress apolipoprotein B (ApoB) secretion in differentiated Caco-2 cells. These conditions could potentially slow the progression of cardiovascular disorders such as atherosclerosis [113].

Although emphasis is typically placed on lipid oxidation, other modified nutritional molecules, such as proteins and nucleic acids, can also affect consumer health. For example, Edalati et al. recently showed that a high intake of ultra-processed food was associated with elevated urinary levels of 8-oxo-dG, a marker of oxidative DNA damage [77]. Current knowledge indicates that carbohydrates and lipids are the main contributors to postprandial oxidative stress, but protein-based meals can also induce ROS generation at a level comparable to that of glucose [114]. Long-term high protein intake has been reported to lead to increased oxidative stress in rats [115,116]. When it comes to individual amino acids, high concentrations of branched-chain amino acids have been shown to induce oxidative stress in peripheral blood mononuclear cells from healthy donors [117]. Similarly, methionine (Met) was reported to increase ROS generation in a murine colitis model, which was alleviated when the Met dietary content was restricted [118]. In contrast, increased intake of dietary cysteine (Cys) was demonstrated to boost GSH levels and therefore cease the postprandial disruption of redox homeostasis in rats fed a high-sucrose diet [119].

In terms of the impact of proteins on postprandial oxidative stress response, meat is the most discussed protein-rich food. However, this debate extends beyond the protein content. Meat is a complex matrix, and besides proteins (including protein carbonyls), it also contains haem and labile iron, both of which may initiate lipid peroxidation and the formation of lipid peroxidation end-products such as MDA, 4-HNE, or oxysterols [120,121]. The impact of repeated exposure of gastrointestinal epithelial cells to these mutagenic and carcinogenic lipid-oxidation products, followed by their absorption and potentially harmful effects on other tissues, has been discussed in the scientific literature [121,122]. In addition to lipid-derived DNA-reactive compounds, other potentially carcinogenic components may be found in processed meat, including (i) heterocyclic aromatic amines, (ii) nitrosamines, (iii) polycyclic aromatic hydrocarbons and (iv) N-glycolylneuraminic acid [123], some of which are formed in radical-mediated reactions. Recently, it has been reported that thermal processing of different types of foods, including meat (specifically beef and pork), resulted in high, temperature-dependent damage to dietary DNA. Once these chemically modified meat-borne nucleobases became incorporated into host DNA, they contributed to endogenous DNA damage in cultured human cells (HeLa, MCF-7, HEK293, and SW620) as well as in cells of the small intestine of rodents that orally consumed such model oxidatively damaged polydeoxynucleotides [124].

In addition to high sugar and lipid loads, whose impact on redox homeostasis has already been mentioned, processed foods commonly consumed within the WD are also rich in food additives, such as certain preservatives, artificial sweeteners, sensory enhancers, or colourings, some of which are also reported to promote oxidative stress [125,126]. Although often neglected, another dietary oxidant is $H_{2}O_{2}$, utilized as an antimicrobial and sterilizing agent for aseptic packaging of fruit juices and milk products. Residual amounts of $H_{2}O_{2}$ may remain inside the package after sealing. Moreover, in several countries (for example, Canada, New Zealand, and the USA), $H_{2}O_{2}$ can be applied as a bleaching agent in foods such as wheat flour or edible oils [127]. Accordingly, to avoid such oxidative challenges, the US Food and Drug Administration set the threshold of the maximum amount of residual $H_{2}O_{2}$ to be present in foodstuffs not to exceed 0.5 ppm [128].

### 3.2. Alcohol Consumption

Ethanol can be naturally produced and absorbed in the GI tract by the host microbiota or during the degradation of threonine, deoxyribose phosphate, and β-alanine, forming acetaldehyde, which is then metabolized in the liver [129]. However, major exposure to ethanol results from consumption, mainly in the form of alcoholic beverages. According to the World Health Organization Global Status Report on Alcohol and Health 2018 [130], in 2016, global alcohol consumption was estimated at 6.4 litres of pure alcohol per person per year, which is equal to the consumption of approximately 1 litre of wine per week. The highest alcohol intake was observed in Europe, with an average of 12–14 litres and 15 litres in Western and Eastern European countries, respectively. According to Eurostat, 8.4% of the European Union population consumed alcoholic beverages daily in 2019 [131]. Furthermore, the COVID-19 pandemic increased the frequency of drinking behaviours due to psychological distress [132,133,134], which has already been shown to lead to a greater burden of alcohol-associated liver diseases and premature mortality [135].

The main part of ethanol metabolism occurs in the liver and involves oxidation reactions catalysed by alcohol dehydrogenase and acetaldehyde dehydrogenase with, respectively, acetaldehyde and acetate as the reaction products. These processes increase the ratio of the reduced to the oxidized form of nicotinamide adenine dinucleotide (NADH/NAD^+^) in mitochondria, which may lead to decreased NEFA oxidation and, thus, accumulation of intracellular lipids [136,137]. Ethanol can also be metabolized by cytochrome P450 2E1 (CYP2E1), the activity of which is significantly increased in alcohol consumers. Since CYP2E1 possesses NADPH oxidase activity, following alcohol consumption, CYP2E1 contributes to the elevated generation of $O_{2}^{•-}$ and $H_{2}O_{2}$ [138]. Both pathways of ethanol metabolism (involving alcohol dehydrogenase or CYP2E1) are implicated in the development of alcohol-induced liver disease [137,139]. Ethanol consumption can also lead to alcohol-induced oxidative disruption of the intestinal barrier via upregulation of iNOS and, thus, excessive generation of NO [140]. These processes implicated in alcohol metabolism can affect redox homeostasis either by altering the NADH/NAD^+^ ratio in the mitochondria or by increasing ROS generation.

### 3.3. Environmental Pollutants Entering the Food Chain

Industrialization of the modern world has made it virtually impossible to avoid exposure to various man-made chemicals that are released into the environment. Humans are surrounded by environmental pollutants in everyday life, which is not insignificant for health [141,142,143,144]. Among these pollutants are those recently recognized as oxidative stress-inducing exogenous factors, which include endocrine disruptors present in foodstuffs (e.g., bisphenols or some pesticides). These “hormone mimics” have been implicated in the development of several of the previously mentioned health-threatening complications.

Bisphenol A (BPA) is a synthetic compound used in the production of packaging materials for foods and beverages, particularly in the inner coating of metal cans and plastic bottles. It has been reported that BPA starts to migrate into foodstuffs at the stage of food production, and this release continues during other stages of the food supply chain, such as handling, packaging, and transportation of food products. The highest concentrations of BPA in foodstuffs have been reported in products stored in plastic bottles (e.g., vegetable oils) and canned products (e.g., canned fish) [145]. BPA is classified in the first category of Endocrine Disruptive Chemicals, meaning there is sufficient evidence that it can exert detrimental effects on the human reproductive system. Moreover, it may be also implicated in the development of cancer, T2D and obesity [146]. One of the mechanisms by which BPA may contribute to health impairment is the deregulation of redox homeostasis. BPA is metabolized by cytochrome P450 to BPA 3,4-quinone, which was shown to be a strong oxidant and inducer of the ROS-generating enzyme—XO [147]. Exposure to BPA was reported to reduce total antioxidant capacity and activity of antioxidant enzymes in numerous tissues in vivo [146]. The systematic review, including 20 animal studies, revealed that BPA could markedly decrease the abundance of GSH and antioxidant enzymes such as catalase (CAT), glutathione peroxidase (GPx), and superoxide dismutases (SOD), as well as cause a significant increase in MDA levels [148]. The markers of oxidative stress were also found to be positively correlated with serum levels of BPA and its analogues in people (*n* = 353) who were highly exposed to this chemical [149].

Another recently identified redox homeostasis disruptor is the widespread usage of plastics. Microplastics (MPs) in marine and terrestrial environments, even in utero, have become notorious contaminants of the environment [150,151]. The annual consumption of MPs in the American population was estimated to reach up to 93,000 MP particles per person, considering that bottled water was the only source of drinking water [152]. The evidence documenting the impact of MPs on redox homeostasis in vitro and in vivo is growing; however, research on this subject is still in its infancy [153]. For example, MPs were shown to increase the level of MDA and to decrease the activity of SOD, CAT, and GPx in cardiac tissue, which contributed to the occurrence of cardiotoxicity in rats [154]. Numerous studies have shown that MPs can accumulate in the gut and liver of aquatic organisms. MPs were found to cause inflammation, alter lipid and energy metabolism, and increase the activity of CAT and SOD in response to oxidative stress in the liver of zebrafish [155]. Another study on zebrafish showed that MPs-induced ROS generation decreased the abundance of antioxidant enzymes and apoptosis via p53 signalling [156]. Consequently, the impact of MP bioaccumulation in foods on human health is also currently widely debated. Although available information is still too limited to draw any hard conclusions [152,157,158], animal studies point to the serious risk of ROS-associated complications also in humans exposed to MPs.

Pesticides are other redox homeostasis disruptors, which have been known for some time. These chemicals are extensively used in heavily industrialized agriculture and, therefore, they contaminate not only foodstuffs but also water and soil. One of the studies found pesticide residues in almost 40% of tested samples of fruits and vegetables available on the Polish market. The highest amounts of pesticide levels were found in gooseberry, apples, grapes, and black currant among fruits, and celeriac, tomato, sweet pepper, and Peking cabbage among vegetables [159]. Numerous studies showed that agricultural workers chronically exposed to pesticides had significantly elevated levels of oxidative stress biomarkers, indicating lipid peroxidation and DNA damage [160,161,162]. Not surprisingly, oxidative stress is considered to be one of the mechanisms by which pesticides exert detrimental effects on human health [160,163]. For example, organophosphates (OPs), one of the most widely used pesticides worldwide, were shown to affect redox homeostasis in rat tissues such as the liver and brain. Exposure of rats to OPs significantly decreased GSH levels in liver and brain tissue, with the concomitant increase in GSSG levels [164]. Interestingly, the GSH/GSSG ratio was not as much disrupted in the rats, which were fed with a mixture of antioxidative vitamins (A, C, and E) for 15 days before exposure to OPs, compared to vitamin-unfed animals [164]. Another study showed that pesticides may lead to mitochondrial dysfunction, as exposure to OPs markedly decreased CoQ_10_ levels in human neuroblastoma cells, as well as reducing the activity of ETC and citrate synthase [165]. Exposure to OP pesticides in utero caused increased levels of MDA in the liver and kidney of female Wistar rats as well as in the offspring. The observed changes persisted from weaning till adulthood, which implied the possible transgenerational adverse effects of pesticide exposure [166]. OPs were also shown to induce inflammation via increased levels of, e.g., nuclear factor kappa-light-chain-enhancer of activated B cells (NF-κB), tumour necrosis factor alpha (TNF-α) and COX-2 as well as increased ROS generation, and DNA damage in human epithelial ovary cells in vitro [167]. Not surprisingly, exposure to pesticides has been related to the development of human diseases such as cancer, allergies, or infertility [168].

## 4. ROS as Inducers of Oxidative Damage to Biomolecules

### 4.1. Oxidation of Nucleic Acids

${\mathrm{HO}}^{•}$ is the major ROS responsible for oxidative damage of DNA, as it can react with its all major components, i.e., purine/pyrimidine bases and deoxyribose. Three main types of oxidative DNA damage can initiate mutations: (i) formation of so-called oxygen DNA adducts, i.e., products of oxidation of nucleobases, (ii) generation of single- and double-strand breaks, and (iii) formation of DNA-protein cross-links as well as DNA adducts with lipid peroxidation products [169]. Among DNA nucleobases, guanine is the most prone to oxidation due to its lowest oxidation potential [170]. The attack of ${\mathrm{HO}}^{•}\;$on 2′-deoxyguanosine results in the formation of 8-hydroxy-2′-deoxyguanosine (8-OH-dG), which together with 8-hydroxyguanine (8-OH-G) and the products of their subsequent oxidation to 8-oxo-(d)G are the most-studied modifications and most frequently assayed markers of oxidative DNA damage [171]. Except for the nucleus, around 1% of DNA is also contained in the mitochondria. Due to its location close to the ETC, mitochondrial DNA (mtDNA) is exceptionally prone to oxidative damage. In addition, mtDNA is particularly susceptible to damage, as it does not contain histones. To make things worse, the mitochondrial repair-system capacity is very limited. For these reasons, mtDNA is notably more prone to oxidation than genomic DNA (gDNA). Indeed, it has been reported that the level of 8-oxo-dG can be even 10–15-fold higher in mtDNA than in gDNA [172,173,174]. Similarly to mtDNA, RNA molecules are also more susceptible to oxidation than DNA, with 8-oxo-G being the most prevalent modified ribonucleoside [175].

All the mentioned chemical modifications of nucleic acids are known to lead to mutagenic and toxic effects. In humans, oxidative DNA damage has long been considered a promoter of cancer and also neurological diseases as well as being implicated in the process of ageing. As mentioned earlier, dietary DNA found in processed foods can be an important, but still not-studied, source of modified nucleobases, which after incorporation into the consumer’s genome may pose health risks as described above.

### 4.2. Lipid Peroxidation

Lipid peroxidation is a process of oxidative damage of unsaturated FAs that is initiated by ROS, especially ${\mathrm{HO}}^{•}$, and further propagated by chain reactions. This cascade begins with an abstraction of the hydrogen atom from the methylene group in a structure of unsaturated FA, especially PUFAs, by free radical attack. As a result, the alkyl radical $\left(R^{•}\right)$ is formed, which can further react with molecular oxygen, leading to the formation of lipid peroxyl radical (${\mathrm{LOO}}^{•}$). The latter is highly reactive, and is able to abstract the hydrogen atom from the adjacent unsaturated FA, forming lipid hydroperoxide (LOOH), and next the alkyl radical, or can undergo cyclization with the formation of endoperoxide [176]. The sequence of propagation reactions goes on continuously, unless the formed lipid radicals react either with each other or with antioxidant molecules, which terminates the chain of reactions by the formation of stable non-radical products [177]. The end products of lipid peroxidation can be divided into primary products (lipid hydroperoxides), which can further decompose into secondary products (e.g., lipid hydroxides, ketones, epoxides, or furans). Moreover, as a result of C-C bond cleavage, various truncated oxidized lipids and low-molecular aldehydes (e.g., MDA or 4-HNE) can be formed. MDA, 4-HNE, and F2-isoprostanes (prostaglandin-like substances that can be formed in the course of non-enzymatic oxidation of arachidonic acid) constitute the most-studied end products of lipid peroxidation. All these metabolites are currently used as biomarkers in oxidative stress assessments [178]. Furthermore, electrophilic products of lipid peroxidation can also initiate the formation of various adducts formed by irreversible conjugation with nucleophilic residues of biomolecules such as nucleic acids, cell membrane phospholipids, or proteins. The formed adducts constitute so-called advanced lipoxidation end products (ALEs) [179].

The effects of products of lipid peroxidation on cell function are detrimental, since they may disrupt cell membranes and induce alterations in their fluidity and permeability as well as functionality, at both lipid and protein levels [110]. The structure and function of biological membranes depend on their constituents, namely, lipid classes and FA composition. The main components of animal cell membranes include phospholipids, glycolipids, and cholesterol, all of which are prone to oxidative modifications [177]. PUFAs present in phospholipids and glycolipids, as already mentioned, are exceptionally susceptible to non-enzymatic lipid peroxidation due to multiple unsaturated bonds present in their structure [180]. Also, cholesterol can undergo autooxidation to oxysterols, the process stimulated by free radicals [181]. However, it should be mentioned that lipid oxidation is not only a detrimental event. For example, enzymatic oxidation of PUFAs catalysed by LOXs, COXs, and cytochrome P450 monooxygenases leads to the formation of lipid mediators, so-called oxylipins, involved in biological processes such as inflammatory and pain responses, vascular tone, or blood coagulation [182]. Changes in circulating oxylipin profiles have been observed in numerous disorders such as cardiovascular disorders, obesity, metabolic syndrome, liver diseases, T2D, neurological disorders, or infections [182]. As mentioned earlier, one of the commonly used biomarkers of oxidative stress are F2-isoprostanes. These prostaglandin-like lipid mediators are formed as a result of non-enzymatic peroxidation of PUFAs under oxidative stress conditions [183]. However, recent studies showed that isoprostanes and neuroprostanes delivered from n−3 PUFAs including EPA and DHA, respectively, could exhibit anti-inflammatory or anti-atherosclerotic properties [183]. Interestingly, the ratio of n−6 to n−3 PUFA oxidation determined based on isoprostanoid profiling was proposed as a biomarker for metabolic complications in obesity [184]. In turn, the oxidation of cholesterol to oxysterols catalysed by cholesterol hydroxylases present in mitochondria or ER is involved in the elimination of excess cholesterol from the human organism [185].

### 4.3. Oxidative Modifications of Proteins

Due to their abundance in biological systems, proteins may be considered the major targets for oxidation [186]. These biomolecules can be attacked by almost any type of ROS. Similarly to the lipid peroxidation process, proteins can also undergo peroxidation, starting from carbon-centred radicals that can be further converted to protein peroxyl radicals and then to protein hydroperoxides [187]. The main carbonyl protein oxidation products include glutamic semialdehyde (a product of arginine oxidation), aminoadipic semialdehyde (a product of lysine oxidation), 2-pyrrolidine (a product of histidine oxidation), and 2-amino-3-ketobutyric acid (a product of threonine oxidation) [188]. Additionally, *ɣ*-glutamyl semialdehyde and aminoadipic semialdehyde are the main metal-catalysed protein oxidation products, which can reach 55–100% of the total carbonyl group content [189]. Carbonylated proteins can also be generated upon reaction with the products of lipid peroxidation, which form the already mentioned ALEs, or AGEs [190]. Another type of irreversible protein oxidative modification is tyrosine nitration, which arises upon the reaction of RNS with tyrosine residues [49]. Oxidative modifications of proteins force changes in their conformation, thereby inducing intramolecular crosslinking, aggregation, or fragmentation, which may finally be responsible either for their diminished or completely abolished enzymatic activity and, as such, disrupted protein function. Since the increased quantity of carbonylated proteins has been observed in numerous diseases, their assessment is widely used as a biomarker of oxidative stress [191].

Among amino acids, the most prone to oxidation are Cys and Met. However, unlike other amino acids, their modifications are reversible (at least to some extent). This feature is essential for the ability of these amino acids to sense any changes in cellular redox status. Not surprisingly, Cys and Met govern cellular thiol-redox signalling, which seems to be of the uttermost importance in terms of the maintenance of redox homeostasis [192,193]. The thiol group of Cys can adopt as many as six different oxidation states: the thiol group is the lowest oxidation state (−II), which, after oxidation (or glutathionylation) of two Cys, can form a disulfide bond with an oxidation state −I. The thiol group can be also oxidized by ROS to sulfenic (–SOH, oxidation state 0), sulfinic (–SO_2_H, oxidation state +II), or sulfonic acid (–SO_3_H, oxidation state +IV). Only the latter modification is considered to be irreversible, and its presence has been associated with many diseases (e.g., T2D, Alzheimer’s, or heart diseases) [194]. In the case of Met, this amino acid can be reversibly oxidized to methionine sulfoxide (MetO), which may be reduced back to Met by methionine sulfoxide reductases (MSRs) [195]. MetO can be further oxidized to methionine sulfone (MetO_2_), a modification considered irreversible and probably relevant only under pathophysiological conditions [196].

## 5. Antioxidants and the Maintenance of Redox Homeostasis

**Figure 3:**
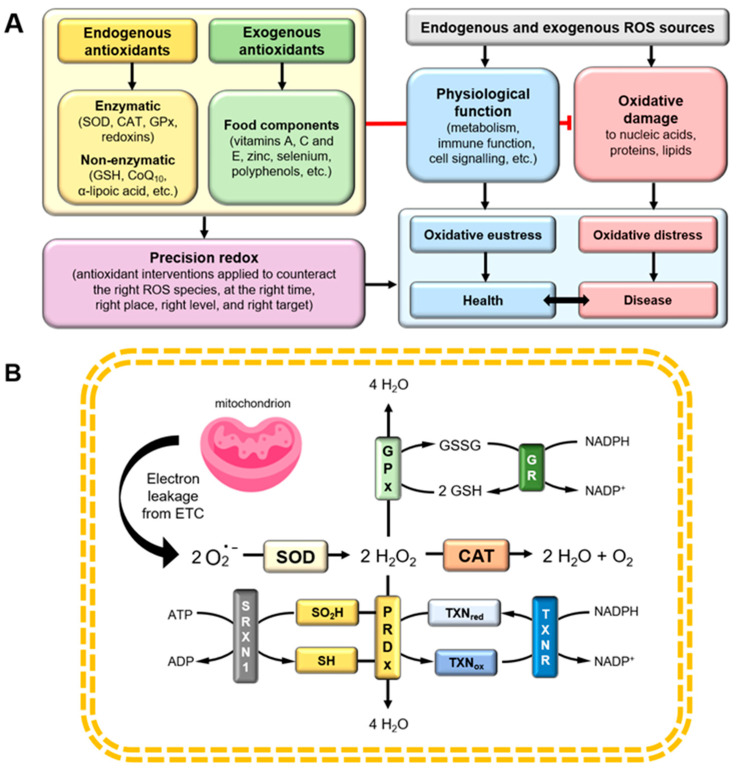
(**A**) Overview of the components that constitute the core of redox homeostasis and which therefore may affect both physiological functions and the development of pathophysiological processes. Precision redox strategy points to crucial factors in designing successful redox-based therapies. Illustration inspired by the work of Sies et al. [10]. (**B**) Concerted action of major enzymatic and non-enzymatic factors constituting endogenous antioxidant system. Electron leakage from the electron transport chain (ETC) serves as an example of endogenous source of reactive oxygen species (ROS). Abbreviations: CAT, catalase; GSH, glutathione; GSSG, glutathione disulfide; GPx, glutathione peroxidase; GR, glutathione reductase; PRDX, peroxiredoxins; SOD, superoxide dismutase; SRXN1, sulfiredoxin 1; TXN, thioredoxin; TXNR, thioredoxin reductase.

ROS and oxidative stress are integral parts of aerobic life, therefore it is not surprising that organisms “developed” systems of antioxidant protection against excessive amounts of oxidants to maintain the redox homeostasis within a cell. According to Le Gal et al., redox homeostasis can be understood as the maintenance of a stable redox status within a dynamic cellular milieu, owing to “(…) *a highly responsive system that senses changes in redox status and realigns metabolic activities to restore redox balance*” [197]. If endogenous systems of antioxidant protection occur to be insufficient or ineffective, they must be supported by exogenous reducing substances derived from dietary sources [198,199]. The overview of the components that constitute the core of redox homeostasis, and which therefore may affect both physiological functions and the development of pathophysiological processes, is presented in Figure 3A.

In the case of food components, an antioxidant is described as a substance that, once consumed, “*delays, prevents or removes oxidative damage to a target molecule*” [200,201]. In a chemical sense, antioxidants (or reducing agents) donate electrons to oxidizing agents, e.g., free radicals, which gain electrons. As a result, oxidants undergo reduction, while antioxidant molecules are transformed into their oxidized forms [202]. The ability of a compound to accept electrons under standard conditions is defined by the *standard reduction potential (E^0^)*. The lower the value of *E^0^*, the stronger the antioxidant activity, as the compound is more likely to donate rather than accept electrons. Most of the data found in the scientific literature refer to the values of the *formal reduction potentials E^0^′*, i.e., determined at pH 7.0 or 7.4, as they are recognized to be more relevant in terms of biological systems [203,204]. Besides thermodynamics, the reactivity of redox-active compounds is also determined by the kinetics of the redox reactions in which they are involved [205].

As a matter of fact, the systematic classification of antioxidants does not exist. They can be assigned to various groups according to, e.g., their origin (natural, synthetic), source (exogenous, endogenous), solubility (hydrophilic, lipophilic), size (low or high molecular weight), or activity (enzymatic, nonenzymatic) [206]. The proposed mechanisms of antioxidant activity include the following: (i) sequestration of free radicals, (ii) chelation of transition metals, (iii) termination of chain reactions, (iv) repair of the oxidatively damaged biomolecules and (v) enhancement of the activity of endogenous antioxidant enzymes. Furthermore, antioxidants can be considered as the first (antioxidant enzymes), the second (endogenous low-molecular-weight antioxidants), or even the third (antioxidant enzymes involved in the repair of oxidative damage) line of antioxidant defence [207,208]. These protective networks correspond to the strategies of antioxidant defence, named as (i) prevention, (ii) interception, (iii) repair and (iv) adaptation [209]. The aim of preventive measures is, just as this term implies, to avert the generation of ROS primarily at the source, while interception is about the neutralization of ROS evading the protective mechanisms [10,210]. Repair refers to the ability of certain enzymes, such as methionine sulfoxide and cystine reductases, to contribute to a reversal of oxidative damage of biomolecules by reduction of the oxidized residues of amino acids in proteins [211,212]. Such reversible redox modifications are also fundamental for redox signalling pathways [15,192,193]. Finally, adaptation processes sustain the expression of antioxidant proteins to improve resistance to oxidative stress [12]. From a physiological point of view, it is also worth mentioning methaemoglobin reductase (also known as cytochrome *b*5 reductase), which plays a crucial role in maintaining the balance between haemoglobin and its oxidized form—methaemoglobin. Methaemoglobin reductase facilitates the reduction of methaemoglobin back to haemoglobin by transferring electrons from NADH or NADPH to methaemoglobin, thus restoring its oxygen-carrying capacity [213].

Antioxidant enzymes constitute the most effective endogenous antioxidative defence, since their cellular abundance (estimated at around 3% of the total cellular protein content) as well as high reaction rates, ensures effective ROS neutralization [214]. Numerous enzymes contribute to the maintenance of redox homeostasis; however, CAT, SODs, and GPxs are considered the major ones (Figure 3B). CAT is the first-discovered enzymatic antioxidant and the major enzyme responsible for the removal of $H_{2}O_{2}$ from the cellular milieu. It is expressed in all major tissues in the human body, with the highest activity in the liver, kidney, and red blood cells [215]. With a catalytic rate of ∼10^7^ M^−1^·s^−1^, it has been estimated that this enzyme can convert around 6 × 10^6^ molecules of $H_{2}O_{2}$ into water molecules and oxygen every minute [216]. SOD, which is another ubiquitous enzyme, occurs in three isoforms in mammalian cells, predominantly cytosolic, but also present in the mitochondrial intermembrane space [217]; SODs catalyse the reaction of $O_{2}^{•-}$ dismutation to $H_{2}O_{2}$ and oxygen at a rate of ∼2 × 10^9^ M^−1^·s^−1^, which is ∼10^4^ times more efficient than spontaneous dismutation. Moreover, under homeostatic conditions, the abundance of SODs in cells is at least 5 orders of magnitude higher than that of $O_{2}^{•-}$ [218,219].

GPxs catalyse the reduction of $H_{2}O_{2}\;$and hydroperoxides with the release of a water molecule or corresponding alcohols at the expense of GSH, which becomes oxidized to GSSG. The rate constant of these reactions is estimated to be ∼10^7^ M^−1^·s^−1^ [220]. Reduction of GSSG back to two molecules of GSH is catalysed by glutathione reductase (GR), which utilizes flavin adenine dinucleotide (FADH_2_) or NADPH as cofactors. There are eight mammalian GPxs, five of which require selenocysteine (SeCys) in their catalytic centre to perform their functions (GPx1-4 and 6), while the other three (GPx5, 7, and 8) are Cys-dependent and do not possess a GSH-binding domain.

Other enzymes indirectly involved in the control of redox homeostasis include peroxiredoxins (PRDXs), thioredoxins (TXNs), and sulfiredoxin (SRXN1). Peroxiredoxins constitute another group of thiol-dependent enzymes that catalyse the reduction of $H_{2}O_{2}$, hydroperoxides, and ${\mathrm{ONOO}}^{-}$. They catalyse reduction reactions at a rate of ∼1–4 × 10^7^ M^−1^·s^−1^, but, unlike CAT and GPx, they do not require cofactors to perform their functions. Based on the structure of their catalytic centre, PRDXs can be further subdivided into typical 2-Cys PRDXs1-4, atypical 2-Cys PRDX5, and 1-Cys PRDX6. Typical 2-Cys PRDXs are most ubiquitous in the cellular milieu. These enzymes perform a catalytic function with the aid of two conserved Cys: one of them is called catalytic or peroxidatic Cys (Cys_P_), and the other one is resolving Cys (Cys_R_). Upon reduction of hydroperoxides, Cys_P_ undergoes oxidation to sulfenic acid, which can be subsequently further oxidized to sulfinic acid. Acquirement of this form results in enzyme inactivation. SRXN1 is indispensable for typical 2-Cys PRDXs reactivation, because, as shown in Figure 3B, it reduces sulfinic to sulfenic acid of Cys_P_ [221,222]. This process is slow and requires ATP, which suggests that, besides providing protection against oxidative damage, SRXN1 contributes to the regulation of $H_{2}O_{2}$-mediated cellular signalling [15,223]. The sulfenic acid group restored by SRXN1 can be further reduced to a thiol group with the aid of the TRX system, which, besides TXN, also involves thioredoxin reductase (TXNR) and NADPH as a cofactor [224]. Due to its disulfide reductase activity, the TRX system is considered one of the major antioxidant enzymatic systems, which greatly contributes to the maintenance of the thiol–disulfide balance [225].

TXNs are small reductases that participate in the reduction of disulfide bonds in target proteins such as the aforementioned PRDXs, but also ribonucleotide reductase and MSR [226]. There are three isoforms of TXN: (i) mainly cytosolic TXN1, which can be transferred to the nucleus, (ii) mitochondrial TXN2, and (iii) TXN3 specific for spermatozoids [227]. Among them, TXN1 is the most-studied one, and is usually referred to as TXN. Experimental studies, using insulin as a preferred substrate for TXN (isolated from *Escherichia coli*), have revealed that a rate constant for TXN action at pH 7 is 10^5^ M^−1^·s^−1^ at pH 7, which is 5 orders of magnitude higher compared to a well-known reductor of thiol groups—dithiothreitol [228]. The rate constant was shown to be even higher (10^6^ M^−1^·s^−1^) for the reduction of the disulfide in the oxidized ribonucleotide reductase [229]. The mechanism of TXN activity involves two Cys residues present in a conserved sequence of amino acids (-Cys_32_-Gly-Pro-Cys_35_-) in this protein active site [230]. One of these Cys (Cys_32_), in its thiolate form, serves as a nucleophilic agent and initiates an attack on the target protein, which results in the formation of a transient state involving a covalently linked mixed disulfide bond. Afterward, the other Cys (Cys_35_), in its deprotonated thiolate form, initiates an attack on the formed disulfide bond, which finally leads to the generation of two reduced thiol moieties in the target protein and a disulfide bond between the two Cys from the active site of TXN [225,229]. The oxidized form of TXN needs to be reactivated by TXNR (each isoform has a corresponding TXNR). Mammalian TXNRs are flavoenzymes, which function as homodimers [231]. Each monomer contains a FAD domain, a pyridine nucleotide-binding domain capable of binding a single molecule of NADPH, and a pair of catalytically active Cys residues situated next to the isoalloxazine ring of FAD. The reduction of the oxidized form of TXN, catalysed by TXNR, is based on a bimolecular nucleophilic substitution and starts with the transfer of electrons from NADPH, serving as a source of reducing equivalents, to FAD. Then, the electrons are transferred from FADH_2_ to a pair of Cys residues situated in an N-terminal active site, followed by a transfer to a pair of Cys and SeCys residues situated in the C-terminal active site of the other TXNR subunit, finally reaching the disulfides in the oxidized TXN [232]. Except for TXN, TXNRs can also reduce other substrates such as, for example, dehydroascorbate, α-lipoic acid (LA), ubiquinone, or cytochrome C [225].

Glutaredoxins (GRXs) are a class of enzymes with thiol–disulfide oxidoreductase activity. Together with GSH and NADPH-dependent GSH reductase, they comprise the GRX system, in which the electrons are transferred from NADPH to GSH reductase (GR), GSH, and ultimately to one of the glutaredoxins (GRX1-3, GRX5-6) [224]. GRXs share functional similarities with TXNs; however, a key distinction between these classes of enzymes lies in GRX’s ability to be reduced back to its reduced state by GSH. Moreover, GRXs can be divided into two groups, based on the number of Cys residues in their active site: (i) enzymes (e.g., cytosolic GRX3 and mitochondrial GRX5) containing only one Cys present in the active site, which catalyse deglutathionylation reactions via a monothiol mechanism, and (ii) those with two Cys in the active site, which follow the dithiol mechanism [233]. Apart from their clear thiol-redox-related properties, GRXs also function as hydrogen donors for ribonucleotide reductase (which is also true for TXNs) as well as exhibiting a dehydroascorbate reductase activity [234].

## 6. Efficacy of Antioxidants

The first indications indirectly suggesting the beneficial effects of antioxidants on human health emerged from epidemiological observations, in which the increased intake of foods rich in flavonoids or vitamin E was inversely associated with the incidence and mortality from CVDs [235,236,237,238]. Subsequently, in numerous specifically designed preclinical in vitro and in vivo studies, dietary antioxidants showed the ability to delay or inhibit lipid peroxidation or LDL oxidation, both of which are known to contribute to the development of atherosclerosis [239]. In 2018, the systematic review and meta-analysis summarizing 69 prospective observational studies concluded that the increased intake of vitamin C, carotenoids, and α-tocopherol was inversely correlated with the incidence of CVD, cancer, and all-cause mortality [240]. Consequently, since oxidative stress frequently accompanies numerous human diseases, such as CVD, cancer, neurodegenerative and metabolic disorders, great expectations were set for chemopreventive strategies, antioxidant-based therapies in particular [2,241,242]. Disappointingly, these promises failed to be fulfilled in human clinical trials [243]. Neither studies verifying the pro-health efficacy of antioxidative vitamins (A, C, and E), nor those applying vitamins from the B group, minerals (such as zinc, selenium), or β-carotene, were successful [244]. For example, the meta-analyses of randomized controlled trials published in 2009 and 2013 by Myung et al. concluded that there is no clinical evidence for the efficacy of antioxidants such as vitamin A, E, and β-carotene in cancer prevention, nor the prevention of CVDs [245,246]. Since different issues concerning the efficacy of antioxidants in clinical trials have been thoroughly discussed elsewhere [206,216,247], in this review only a few points worth reminding of will be raised.

There are three issues, which could have contributed to the observed failures of antioxidants in clinical trials. Firstly, most clinical trials investigated the efficacy of indispensable nutrients such as vitamins, whose functions go far beyond being just antioxidants. For example, vitamin C is a necessary cofactor of enzymes involved in tissue regeneration, collagen formation, or biosynthesis of neurotransmitters [248]. Supplementation of such antioxidant nutrients may be expected to be effective when deficiency is diagnosed; however, not necessarily in well-nourished populations. Secondly, antioxidative nutrients were expected to delay the occurrence of diseases if taken in high doses, which also proved to be ineffective. The already mentioned β-carotene taken at doses of 20 or 30 mg/day, which exceeded the recommended daily intake (2–4 mg) several fold, in male smokers and workers exposed to asbestos, increased the risk of lung cancer by 17% and 28%, respectively [249,250,251]. This outcome questioned the use of excessive doses resulting in the endogenous concentrations of nutrients falling beyond the optimal range, which could lead to adverse effects rather than improved health. Last, but not least, it was expected that providing the antioxidative nutrients in the form of a cocktail would exert synergistic, or at least additive, effects. However, such an approach also failed to reduce the risk of, e.g., colorectal cancer (mix of 1 g vitamin C, 25 mg β-carotene, 400 mg vitamin E) or lung cancer (mix of 30 mg β-carotene and 25,000 IU vitamin A) [5,6]. In the light of the current studies, it seems evident that the activity of compounds in a mixture may not necessarily exhibit enhanced function, let alone the unlikely resemblance of such mixtures to the complex food matrix [205,252,253]. These results may be associated with the concept of food synergy, which is based on the idea that the health benefits of whole foods are greater than the sum of their individual nutrients. For more details on this topic, the reader can refer to [205,252,253].

The disappointing outcome denying the health-promoting efficacy of antioxidants stemmed mainly from studies focused on antioxidative vitamins and β-carotene, whose levels in the human organism must be strictly controlled. Nevertheless, this resulted in labelling all antioxidants as ineffective or of questionable protective potency in the human organism. However, besides the discussed antioxidative nutrients, there is a plethora of foodborne antioxidants whose efficacy has not been thoroughly investigated in large-scale controlled human studies. With the current expectations, much focus has been on the Mediterranean diet (MD). The health-promoting effects of MD were first demonstrated in the Seven Countries Study (SCS), which was the first epidemiological project that investigated the relationship between diet, lifestyle, and health. The SCS showed that populations living in Southern Italy and Greece had significantly lower risk of CVD compared to Western countries, such as the United States [254]. These differences were not fully understood at that time; however, it was hypothesized that the Mediterranean nutrition pattern could provide some answers. Indeed, MD is rich in olive oil, herbs, garlic, onion, and other plant-borne foods, which constitute rich sources of plant-derived antioxidants from a group of polyphenols.

According to the Phenol-Explorer database, over 500 different polyphenols are present in foods [255]. As identified by Pérez-Jiménez et al., spices and herbs (e.g., cloves, peppermint) are the richest sources of polyphenols, followed by other food groups (ordered by decreasing polyphenol content): fruits (e.g., darkly coloured berries), seeds (e.g., flaxseed, nuts), vegetables (e.g., black and green olives, green chicory), non-alcoholic beverages (e.g., coffee, black and green tea) and oils (extra virgin olive oil and rapeseed oil) [255]. So far, the evidence for the health-promoting potential of food and diets (especially the ones rich in polyphenols), beyond just MD, has been overwhelming [256,257,258]. The antioxidant activity of polyphenolic compounds stems from their ability to donate a hydrogen atom (proton and electron) from the hydroxyl group, present in the aromatic ring, which forms a phenoxyl radical. Moreover, polyphenols can also chelate transition metal ions and inhibit the activity of ROS-generating enzymes such as NOX, LOX, and COX [259]. As aptly pointed out by Gutteridge and Halliwell [260], the antioxidant “power” of polyphenols can be also attributed to their mild pro-oxidant properties, i.e., the ability to induce the Nrf2-Keap1 system, and hence increase the levels of antioxidant enzymes and boost a pool of a reduced form of GSH [260,261]. The aspect of pro-oxidant properties of polyphenols has gained attention during recent years; importantly, the chemical properties seem to play a leading role in understanding their complex mechanisms of antioxidant/pro-oxidant activity [259,262,263]. An increasing number of human intervention trials evaluating supplementation with polyphenols such as catechins, resveratrol or curcumin, have shown numerous health benefits. Enumeration of all the evidence supporting the efficacy of various antioxidants in the treatment of diet-related diseases is beyond the scope of this review, and therefore we will name just a few examples. A meta-analysis of 6 human trials showed that resveratrol supplementation improved cardiometabolic biomarkers in patients with T2D, and thus could be considered as an adjuvant during pharmacological treatment of this disease [264]. Another meta-analysis of 16 controlled trials concluded that resveratrol supplementation improved metabolic parameters (e.g., levels of glucose and triacylglycerols) in metabolic syndrome patients [265]. A randomized controlled trial involving 240 prediabetic patients showed that 9-month curcumin supplementation successfully prevented the development of T2D [266]. Meta-analysis of 7 human trials showed that curcumin and turmeric consumption improved the blood lipid profile among patients at risk of CVD, suggesting potential benefits of their combination with pharmacological treatment [267]. Finally, a systematic review and meta-analyses concluded that catechin supplementation improved some cardiac health-related parameters (e.g., flow-mediated dilation and pulse wave velocity) [268], and improved the lipid profile in overweight and obese people [269]. Results of another meta-analysis of 46 randomized controlled trials suggested that polyphenols, both as whole food and purified extracts, can exert beneficial effects on some parameters of cardiometabolic health, i.e., LDL cholesterol, total cholesterol, triacylglycerols and systolic blood pressure. Greater effectiveness has been achieved in larger interventions with longer follow-up periods than by the smaller ones [270]. Here, it is of note that in 2020 Crowe-White et al. proposed the first-ever guidelines for consumption of polyphenolic compounds—flavan-3-ols (commonly known as catechins)—at doses of 400–600 mg/day, to support cardiometabolic health. The authors emphasized that these recommendations refer to food-based intake of catechins, but not dietary supplements [271]. On the other hand, the most recent meta-analysis of 40 human clinical trials investigating the efficacy of dietary polyphenols in the management of obesity concluded that polyphenol intake did not lead to clinically advised improvements for obesity, despite statistically significant reduction in BMI, body weight, and waist circumference. However, when combined with regular physical activity, a healthy, balanced diet, and other lifestyle changes, dietary polyphenols can be used as an additional supplement to help manage obesity [272].

Importantly, polyphenols are not the only antioxidants, which may bring benefits. The results of trials that were discussed in this review, in which supplementation with endogenous antioxidants was used, also brought promising results. For example, a meta-analysis of 15 human trials showed that LA significantly reduced levels of MDA, a biomarker of lipid peroxidation, though it did not improve other markers of oxidative stress [273]. Other meta-analyses showed that supplementation with LA improved glycaemic and inflammatory biomarkers (fasting glucose, haemoglobin A1c, C-reactive protein, interleukin 6, and TNF-α) among patients at risk of cardio-metabolic disorders [274,275]. In a meta-analysis of 16 human trials, supplementation with CoQ_10_ was shown to reduce levels of MDA and to increase the activity of SOD as well as total antioxidant capacity [276]. These findings are in line with conclusions from a more recent umbrella meta-analysis of 13 meta-analyses (including 77 trials, in total) [277]. The potential of CoQ_10_ to alleviate oxidative stress could also contribute to the reduced risk of CVD in diabetic patients, which was reflected by lowered total cholesterol and LDL levels, according to the meta-analysis of 12 human trials [278]. A meta-analysis of 50 randomized controlled trials confirmed that CoQ_10_ supplementation significantly improved the lipid profile (i.e., reduced the total cholesterol level, LDL, and triacylglycerols and increased the HDL level) in adults [279]. Another physiologically relevant antioxidant that has been shown to improve oxidative stress parameters is melatonin. A meta-analysis of 12 randomized controlled trials reported that melatonin intake was positively associated with increased total antioxidant capacity (TAC), level of reduced GS, enhanced activity of antioxidant enzymes (GPx, GR, SOD), and decreased level of MDA [280].

## 7. Underestimated Relevance of Redox Chemistry

Cellular redox status is regulated in a spatiotemporal manner. The formal reduction potential of certain redox couples, such as GSSG/GSH, due to, e.g., different pH conditions, depends on their intracellular localization: in mitochondria, it is around −300 mV, from −220 to −260 mV in cytoplasm, from −130 to −153 mV in cellular membranes, −150 mV in ER and −240 mV in lysosomes [247]. This indicates that the basal cellular redox state is compartment-specific and may be either more reducing or more oxidizing, depending on physicochemical parameters and the overall pool of redox-active substances, which underlie the compartment’s role in the maintenance of cellular function. A more-oxidizing milieu is necessary for proper protein folding in ER, while a slightly oxidizing environment in the cytoplasm triggers redox signalling events through changes in the $H_{2}O_{2}$ levels and reversible oxidation reactions of thiol groups present in proteins. For example, the activity of PRDXs can be modulated (or even inhibited) by $H_{2}O_{2}$-driven oxidation of thiol groups localized in the catalytic centres. In yeasts, as well as in mammalian cells, PRDXs were shown to further mediate $H_{2}O_{2}$-dependent redox signalling through redox-sensitive transcription factors such as signal transducers and activators of transcription (STATs), which suggests that antioxidant enzymes are not just so-called “sinks of oxidants”, but they can also actively participate in the regulation of redox signalling [281]. Finally, the redox state can also change periodically, following different stages of the cell cycle [282].

The so-much-feared cellular damage resulting from the elevated levels of oxidizing agents, such as ROS/RNS, occurs only when the homeostasis is disrupted. In the case of tumour-targeted redox therapies, it was shown that low levels of ROS stimulate tumour growth, whereas ROS overload leads to cell cycle arrest and apoptosis. In the case of antioxidants, they can either prevent the initiation of carcinogenesis by curbing oxidative stress or, in contrast, support different stages of tumorigenesis, as was observed in the case of β-carotene supplementation, which increased the incidence of lung cancer in smokers and workers exposed to asbestos [249,283]. Currently, there is neither a clear threshold set to define proper redox homeostasis nor indications of how much ROS is too much. Beyond redox orchestration for proper cellular function, redox-regulated intracellular instructions can also be altered by circadian rhythms, seasons, external stimuli, and disease development processes, not to mention individual human variability [284]. For instance, in the case of carcinogenesis, the levels of ROS and antioxidants change as the disease progresses. In the initial stages, the antioxidant defence declines with a concomitant increase in ROS production, which results in oxidative DNA damage and an elevated risk of mutations. In more advanced stages of cancer, the levels of antioxidants are elevated to mitigate the toxicity of ROS overgeneration [285]. For example, it has been reported that the decrease in ROS levels caused by vitamin E and NAC supplementation reduced p53 expression levels and induced tumour progression in genetically engineered mice, since apoptosis being under the control of this protein and subsequent tumour cell death could not take place [286]. Therefore, antioxidant supplementation in conjunction with cancer therapies remains a controversial and highly arguable issue [287,288]. The double-sword nature of both ROS and antioxidants is now referred to as the antioxidant paradox [244,289].

## 8. Conclusions

Oxidative stress has been known in biological sciences for decades; however, the understanding of this concept has highly evolved since its foundation in the mid-1980s. Currently, the extended definition covers not only the subject of the oxidation–reduction balance, but also the consequences of altered redox signalling. One of the most significant changes in the discussion on oxidative stress is that it is now understood as the intrinsic component of life; ROS are no longer perceived exclusively as unwanted and deleterious molecules. Nevertheless, the exposure to various factors in everyday life that contribute to the intensified ROS generation are inevitable, and thus the recommendations for antioxidant-rich diets are justified. Though initially it seemed that the more antioxidants the better, currently, the other side of the coin—the risk associated with reductive stress—has emerged. Nowadays, the question about antioxidants seems to be “how much is enough?”. Furthermore, similarly to ROS, the term “antioxidants” also works as an “umbrella” term. Redox-active compounds differ in chemical structure, electrochemical properties, mechanisms of antioxidant activity, and bioavailability, and therefore, efficacy in protecting against excessive load of oxidants that vary in redox properties, inevitably differs as well. These factors make it difficult to expect that such a chemically diverse group of molecules would affect the human organism in the same way, just because all of them possess reducing properties. In light of our current understanding, the past assumptions that any antioxidant would act similarly turned out to be rather naive. Fortunately, the failure of clinical trials to prove the efficacy of some dietary antioxidants (mainly nutritive) in disease prevention has not ceased the research focused on the health-promoting properties of dietary antioxidants. Therefore, new strategies (such as Precision Redox) are being designed, and it is now well understood that their effectiveness may depend on the type of antioxidant (endogenous or exogenous), whose activity could either maintain redox balance (under challenging conditions) or restore it when disrupted (state of oxidative or reductive stress). Precision Redox takes into account human variability in the redox status and should be applied taking into consideration the right chemical species (either oxidants or antioxidants), the right time (regarding dynamic redox fluctuations), the right place (organelle-, cell- or tissue-specific), the right level (the twofold effects of ROS/antioxidants depending on their doses), and the target (the delay of oxidative modifications of macromolecules through personalized antioxidant strategies). There are thus numerous variables that decide whether an antioxidant-based therapy will be successful or not. First of all, such an approach still remains challenging, due to methodological limitations. Neither is there a clear quantitative definition of oxidative stress, and thus no population-based reference range of the “normal” redox state nor standardized methods to successfully approach these gaps in clinical and diagnostic settings. To conclude, more research on antioxidants of either endogenous or exogenous origin is required for the Precision Redox strategy to be successfully implemented.

## Figures and Tables

**Table 1:** The most important endogenous and exogenous sources of reactive oxygen species (ROS) generation [22,23,35,56,57,58,59,60].

Triggers of ROS Production	
Endogenous	Exogenous
**Cytosol** Purine catabolism Auto-oxidation of low-molecular-weight compounds **Endoplasmic reticulum** Xenobiotic metabolism Unsaturated fatty acid biosynthesis Protein folding Thiol oxidase reactions **Mitochondria** Electron transport chain: complex I (sites IF, IQ); complex III (site IIIQo) Monoamine oxidase α-Ketoglutarate dehydrogenase Pyruvate dehydrogenase Glycerol 3-phosphate dehydrogenase The electron transfer flavoprotein: Q oxidoreductase p66shc **Peroxisomes** Fatty acid α- and β-oxidation Ether-phospholipid biosynthesis Glyoxylate metabolism Amino acid catabolism Polyamine oxidation Oxidative part of the pentose phosphate pathway **Plasma membrane** Arachidonic acid oxidation Phagocytic and non-phagocytic oxidative burst	**Physical stressors** UV radiation Ionizing radiation **Chemical stressors** Air pollutants Tobacco smoke Heavy metals Drugs (e.g., paracetamol, doxorubicin) Micro- and nanoplastics **Diet-derived** Oxidized oils Thermally processed meat Pesticides Alcohol **Mental stressors** Negative life changes Catastrophic events Daily hassles Chronic emotional stressors
